# Magnetic Resonance-Based Synthetic Computed Tomography Using Generative Adversarial Networks for Intracranial Tumor Radiotherapy Treatment Planning

**DOI:** 10.3390/jpm12030361

**Published:** 2022-02-26

**Authors:** Chun-Chieh Wang, Pei-Huan Wu, Gigin Lin, Yen-Ling Huang, Yu-Chun Lin, Yi-Peng (Eve) Chang, Jun-Cheng Weng

**Affiliations:** 1 Department of Medical Imaging and Radiological Sciences, and Graduate Institute of Artificial Intelligence, Chang Gung University, Taoyuan 33302, Taiwan; jjwangucla@gmail.com (C.-C.W.); zxc0914@gmail.com (P.-H.W.); 2 Department of Radiation Oncology, Chang Gung Memorial Hospital at Linkou, Taoyuan 33302, Taiwan; 3 Department of Medical Imaging and Intervention and Institute for Radiological Research, Chang Gung Memorial Hospital at Linkou and Chang Gung University, Taoyuan 33302, Taiwan; giginlin@gmail.com (G.L.); b9102091@cgmh.org.tw (Y.-L.H.); jack805@gmail.com (Y.-C.L.); 4 Clinical Metabolomics Core Lab, Chang Gung Memorial Hospital at Linkou, Taoyuan 33302, Taiwan; 5 Department of Counseling and Clinical Psychology, Columbia University, New York, NY 10027, USA; tiramisueve@gmail.com; 6 Medical Imaging Research Center, Institute for Radiological Research, Chang Gung University and Chang Gung Memorial Hospital at Linkou, Taoyuan 33302, Taiwan; 7 Department of Psychiatry, Chang Gung Memorial Hospital, Chiayi 61363, Taiwan; 8 Department of Medical Imaging and Radiological Sciences, Chang Gung University, No. 259, Wenhua 1st Rd., Guishan Dist, Taoyuan 33302, Taiwan

**Keywords:** deep learning, generative adversarial net (GAN), attenuation correction, MR-only simulation, radiotherapy planning, brain tumor

## Abstract

The purpose of this work is to develop a reliable deep-learning-based method that is capable of synthesizing needed CT from MRI for radiotherapy treatment planning. Simultaneously, we try to enhance the resolution of synthetic CT. We adopted pix2pix with a 3D framework, which is a conditional generative adversarial network, to map the MRI data domain into the CT data domain of our dataset. The original dataset contains paired MRI and CT images of 31 subjects; 26 pairs were used for model training and 5 were used for model validation. To identify the correctness of the synthetic CT of models, all of the synthetic CTs were calculated by the quantized image similarity formulas: cosine angle distance, Euclidean distance, mean square error, peak signal-to-noise ratio, and mean structural similarity. Two radiologists independently evaluated the satisfaction score, including spatial, detail, contrast, noise, and artifacts, for each imaging attribute. The mean (±standard deviation) of the structural similarity indices (CAD, L2 norm, MSE, PSNR, and MSSIM) between five real CT scans and the synthetic CT scans were 0.96 ± 0.015, 76.83 ± 12.06, 0.00118 ± 0.00037, 29.47 ± 1.35, and 0.84 ± 0.036, respectively. For synthetic CT, radiologists rated the results as evincing excellent satisfaction in spatial geometry and noise level, good satisfaction in contrast and artifacts, and fair imaging details. The similarity index and clinical evaluation results between synthetic CT and original CT guarantee the usability of the proposed method.

## 1. Introduction

Computed tomography (CT) simulation is a necessary procedure performed after mold customization for every patient who will undergo radiation therapy. It provides information on electron density and geometry. Radiotherapy treatment planning, image reconstruction, and daily treatment guidance are all based on electron density and geometric information provided by CT. Daily treatment is also based on the CT simulation image for image guidance and positioning error correction. Therefore, the most critical issue of MR-only simulation workflows is retrieving this information only through MRI. It can be done by rigid or deformable registration, but errors are inevitable, so high-quality results cannot be expected. Therefore, a CT-independent method should be developed as a better solution.

Magnetic resonance imaging (MR) and CT are both important medical imaging systems for radiotherapy treatment planning. MR is used to segment the tumor contour and volume in radiotherapy treatment planning, and CT is used to calculate the radiation dose. MR imaging-only radiotherapy planning is a novel application. Electron density information typically acquired from CT images is a prerequisite for attenuation correction and radiotherapy treatment planning. However, MR images represent only longitudinal tissue, transverse relaxation times, and proton-density information. To solve this problem, several methods have been developed to produce synthetic CT images for both MRI-only radiotherapy treatment planning and MRI-based attenuation correction (MRAC) [1,2,3,4]. Therefore, the methods that produce accurate attenuation correction on PET–MRI could potentially be applied in MRI-only radiotherapy treatment planning and vice versa. Furthermore, if a single synthetic CT generation method could be used in both applications, there would be no need to use independent sequences or processing pipelines for producing synthetic CT between different systems and modalities. However, a thorough evaluation of the method’s robustness in patients with brain tumors should be performed [5].

MR simulation can provide better sensitivity, specificity, and contrast in soft tissue for image-guided radiation therapy (IGRT) treatment planning. However, a remaining challenge lies in obtaining a reliable X-ray attenuation correction map, which is crucial for the calculation of treatment planning. Many novel strategies [6,7,8] have been introduced to directly estimate bone information for MR imaging-based attenuation correction, including atlas-based methods and image-segmentation-based methods, particularly those using ultrashort echo time and zero echo time (ZTE) approaches [6]. Zero echo time pulse sequences have been used to successfully generate synthetic CT images that can be used for accurate MRAC and MRI-only radiotherapy treatment planning of the brain [9]. Methods based on deep learning have been successfully used to generate synthetic CTs from contrast-enhanced images [10]; however, the effect of contrast agents in synthetic CT generation and their effects on radiation therapy (RT) plan quality have not been studied extensively in an individual study. Although each of these proposed solutions has specific advantages and limitations, the development of rapid and robust MRAC is still currently an unmet need.

Radiotherapy for brain tumors starts at the time of simulation, and the simulation procedure retrieves three-dimensional (3D) images for target delineation and treatment planning. Therefore, magnetic resonance imaging (MRI) has superior soft tissue contrast and improves the target delineation of radiotherapy for brain tumors. Using CT imaging alone, the segmentation of the brain tumor may overestimate the tumor volume. If overestimation can be corrected by MRI, normal tissue damage will be reduced in the future. Unfortunately, MRI cannot be used for radiotherapy planning directly since it lacks electron density information for radiation dose calculation. Although MRI can be registered with CT images and incorporated into radiotherapy planning, this approach is not accurate enough due to misregistration or image distortions [11]. Redundant imaging also increases costs and consumes more time in clinical practice. Developing an MR-only simulation workflow can significantly improve the quality of radiotherapy treatment planning, and time and cost can also be saved.

U-net is proposed by Ronneberger et al. for biomedical image segmentation, named after is the architecture of the model [12]. The structure of u-net is similar to an alphabet “U”. The input side is mainly several layers of CNN-base encoder which can reduce dimensions of input data. On the contrary, the output side is a decoder for expending dimensions. The outstanding design of shortcut connection between encoder and decoder transfer information greatly enhances the performance. Owing to its powerful capacity, the usage of u-net extends further to image translation.

PatchGAN is a classifier for classification tasks [13]. Traditional classifiers present the judgment of input images with a single number, whereas patchGAN does not. The output of patchGAN is a matrix. Each element inside the matrix is the judgment of the corresponding receptive field. That is to say, the mechanism of patchGAN is to separate the original image into different patches and utilize the distinguishing characteristics of all the patches within a matrix. 

Generative adversarial networks (GANs) [14], which are made of MLPs, have achieved great success and have spread explosively since being proposed in 2014. The GAN approach in the method of competition constantly modified the parameters of its MLP. There are many GAN extensions and applications currently, and pix2pix [13] is one of them. U-net [12] of pix2pix has shortcut structures that make information pass through from the encoder to the decoder, producing a more precise pattern. At the same time, the patchGAN of pix2pix forces u-net to its limit on generating pseudoimages.

Currently, many researchers try to solve biomedical imaging issues with deep-learning-based approaches. The latest studies have proven that deep-learning methods are able to learn the nonlinear mapping relationship of the MR domain and CT domain in a two-dimensional framework. Now the issue is pushed to a 3D framework. Dong et al. presented a 3D fully convolutional network to estimate pelvic CT images from MRI data. Liu Y et al. took advantage of 3D-based cycleGAN to convert MR to CT [15,16,17], achieving a great result on 3D-based MRI to CT translation. The advantage of unsupervised-learning cycleGAN is that the dataset where images from two domains are not necessarily paired is easier to prepare. These studies take advantage of a single model to manage their dataset, which is separated into several patches in common. The model of these studies convert a single patch at a time, and all patched predictions are merged at the final stage. The usage of these methodologies is analogous taking a microscope to see a huge object. However, a model has limited capacity to learn all features from a complex 3D-based dataset, and the important features vary from the location of the body. The content of the dataset should be based on the location of the body and each location should have its own corresponding model to deal with. In this paper, we propose a new approach that is expandable to prevent limitations from the model’s learning capacity and to enlarge the image size (resolution) of synthetic CT.

## 2. Methods

### 2.1. Prepare MR–CT Paired Dataset

This study was approved by the Institutional Review Board of Chang Gung Memorial Hospital at Linkou, Taoyuan, Taiwan (No. 202002387B0). All methods were carried out in accordance with relevant guidelines and regulations. Both MRI and CT scans of each participant were acquired at Chang Gung Memorial Hospital at LinKou, Taoyuan, Taiwan. Multimodal MRI examinations were performed on a 1.5T MRI scanner (GE, Boston, MA, USA) with a standard head coil. The three-dimensional T1-weighted gradient-echo sequence (MPRAGE) was obtained with repetition time (TR)/echo time (TE)/inversion time (TI)/flip angle (FA) = 7.91 ms/3.27 ms/450 ms/12°; voxel size = 0.39 × 0.39 × 1.0 mm^3^, and number of average = 2. CT examinations were performed on a GE Light Speed RT16 with a standard brain protocol. Scan parameters were as follows: slice thickness = 1.25 mm of slice thickness, 120 kV of slice thickness, 300 mA of tube current, and 1071 msec of exposure time.

### 2.2. Data Preprocessing

**Figure 1:**
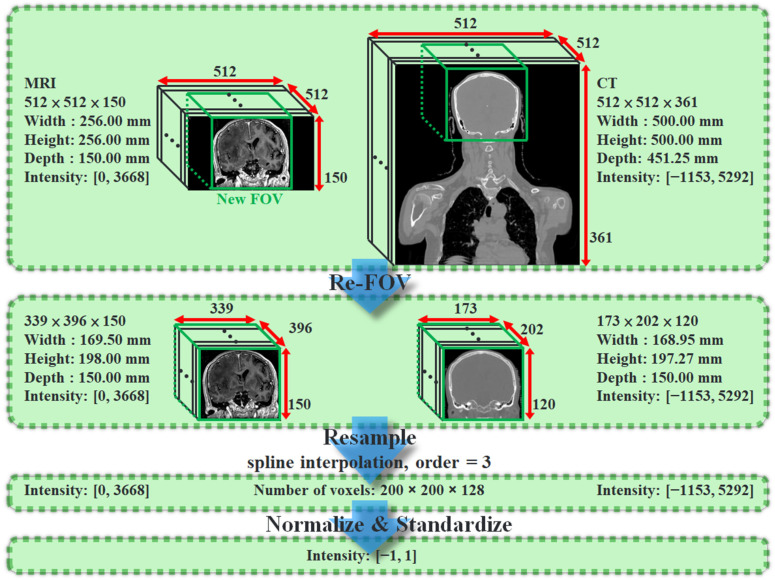
The workflow of data preprocessing. The parameters in Figure 1 come from one of the real cases in the original dataset. The workflow is applied to all pairs of original datasets.

The original dataset has 31 pairs in total, including 26 pairs for training and 5 pairs for tests. To verify the algorithm and make sure the testing dataset covers symptoms in different degrees simultaneously, the five testing pairs are selected. In comparison with the subjects of training pairs, some subjects of the testing pair had wider tumor volumes and others had previously undergone major surgery; they were the outliers of the full dataset. The details of the dataset are listed in Supplementary Materials Table S1. Each image pair is made of an MRI and a CT of a subject. Figure 1 shows the data preprocessing workflow diagram, and Supplementary Materials Table S2 shows the modified image parameters after reFOV and resampling for a few more subjects from the training and test data. MRI and CT are different imaging modalities with different voxel sizes (resolutions), FOVs, window widths, and levels of intensity. Dataset preprocess has 3 stages. In the first stage, we define a new FOV for each pair, and any voxels located outside the new FOV are abandoned. In our original dataset, every MR image has a smaller FOV than CT, so the definition of a new FOV is based on MR. After the re-FOV stage, every paired image has the same FOV but a different number of voxels. In the second stage, we use NiBabel, which is a Python library for medical images, for dataset resampling. The resampling method is spline interpolation with an order of 3. Therefore, each paired image has the same FOV, and the number of voxels of the whole dataset is 200 × 200 × 128. In the third stage, every image was normalized and standardized by Formulas (1) and (2), respectively. The intensity of all images are shifted to −1~1.

(1) $$img^{\prime }=\frac{img-\mu \left(img\right)}{\sigma \left(img\right)}$$

(2) $$\{\begin{array}{c} {img}^{\prime \prime }=2\times \left(\frac{img^{\prime }-m}{d}\right)\\ m=\frac{max\left(img^{\prime }\right)+min\left(img^{\prime }\right)}{2}\\ d=max\left(img^{\prime }\right)-min\left(img^{\prime }\right) \end{array}$$

### 2.3. Patch-Based Datasets

**Figure 2:**
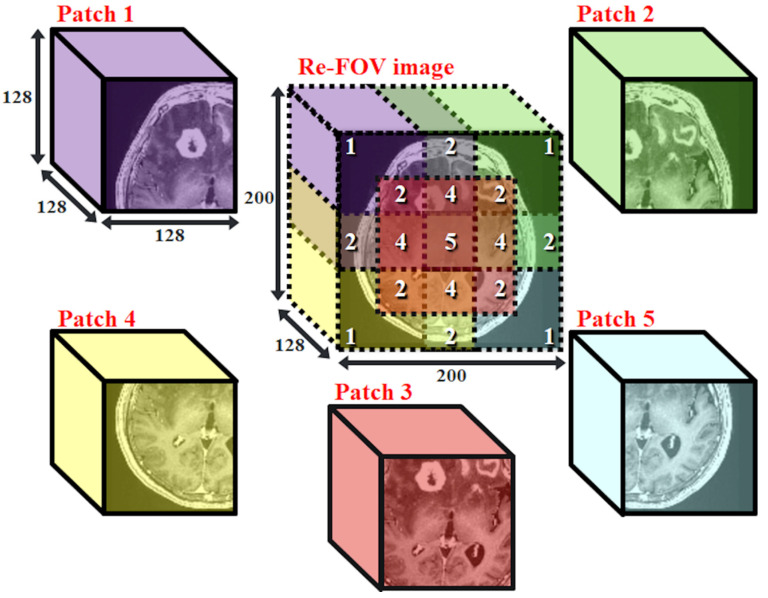
The correlation between five patch and re-FOV image. Patches p1~p5 have their own spatial locations at the coordinates of the re-FOV image. In this paper, we call the collection of the same patch from all subjects the patched dataset.

MRI and CT are medical images with high complexity. To increase the similarity of prediction and prevent trainable parameters from exceeding the limitation of hardware, we obtain 5 different patched datasets, denoted as datasets p1, p2, …… and p5, respectively, from the complete re-FOV dataset. We set the number of voxels to 128 × 128 × 128 for every patch, such that all 3D-pix2pix models can apply with the same architecture. A schematic diagram of patch-based datasets is shown in Figure 2. For example, dataset p1 contains the upper left corner cubes of all subjects in the re-FOV dataset.

### 2.4. Data Augmentation

To overcome the problem of lacking training data, we adopt a data augmentation technique in the training process. Many studies have proven that adopting data augmentation is effective for improving model accuracy for classification tasks [18,19,20]. We rotate the voxel coordinate of every image, and the rotation angle is different at each epoch. The rotate angle along 3 axes is shown in (3) and denoted as ($\theta_{x}$, $\theta_{y}$, $\theta_{z}$). A rotation angle contains a fixed part and a random part. The fixed part is denoted as $\theta_{f}$, and the subscript f means fixed. The random part is denoted as $\theta_{r}$, and subscript r means random. As the iteration increases, the value of $\theta_{f}$ is chosen from 1, −1, 3, −3, 5, −5, 7, and −7, in order. The total 512 combinations are listed as follows:

$$(1+\theta_{r},\;1+\theta_{r},\;1+\theta_{r}),\;(1+\theta_{r},\;1+\theta_{r},-1+\theta_{r}),\;(1+\theta_{r},\;1+\theta_{r},\;3+\theta_{r}),$$

$$(1+\theta_{r},\;1+\theta_{r},-7+\theta_{r}),\;(1+\theta_{r},-1+\theta_{r},\;1+\theta_{r}),\;(1+\theta_{r},-1+\theta_{r},-1+\theta_{r}),$$

$$(1+\theta_{r},-1+\theta_{r},-7+\theta_{r}),\;(1+\theta_{r},\;3+\theta_{r},\;1+\theta_{r}),\;\cdots \cdots ,\;(1+\theta_{r},-7+\theta_{r},-7+\theta_{r}),$$

$$(-1+\theta_{r},\;1+\theta_{r},\;1+\theta_{r}),\;\cdots \cdots ,\;(-7+\theta_{r},-7+\theta_{r},-7+\theta_{r}),\;\mathrm{and}\;(-7+\theta_{r},-7+\theta_{r},-7+\theta_{r}).$$

(3) $$\{\begin{array}{c} \left(\theta_{x},\theta_{y},\theta_{z}\right)=\left(\theta_{f}\left[i\right]+\theta_{r},\theta_{f}\left[j\right]+\theta_{r},\theta_{f}\left[k\right]+\theta_{r}\right),where\;i,\;j,\;k=0,\;1,\;2,\cdots ,7\\ \theta_{f}=\left\{1,-1,\;3,-3,\;5,-5,\;7,-7\right\}\\ \theta_{r}=\left\{\theta |-1<\theta <1\right\} \end{array}$$

### 2.5. Generative Adversarial Nets

Since being proposed by Ian Goodfellow, generative adversarial nets (GANs) have become prosperous in many fields, especially in computer vision (CV). A well-trained GAN is capable of generating pseudoimages that are extremely similar to real images. A GAN is composed of a discriminator and a generator. The task of a discriminator is to judge whether an image is real or not. On the other hand, the generator has entirely opposite goals to the goal of the discriminator. The generator tries to deceive the discriminator such that the discriminator makes the wrong judgment. As the number of iterations increases, the discriminator becomes more capable at judgment, and the generator becomes better at forging. The GANs objective function is shown in (4):

(4) $$\underset{G}{\min}\;\underset{D}{\max}\;V\left(D,\;G\right)=E_{x\sim p_{data}\left(x\right)}\left[\log D\left(x\right)\right]+E_{z\sim p_{z}\left(z\right)}\left[\log\left(1-D\left(G\left(z\right)\right)\right)\right]$$

where the data from the real world are denoted as x; the random noise is denoted as z; the discriminator and generator are denoted as *D* and *G,* respectively, and the synthetic data are denoted as G(z). The distributions of real-world data and random variables are denoted as $p_{data\left(x\right)}$ and $p_{z\left(z\right)}$, respectively. The meaning of subscript $x\sim p_{data}$ is that *x* belongs to $p_{data\left(x\right)}$, and the meaning of subscript $z\sim p_{z\left(z\right)}$ is that *z* belongs to $p_{z\left(z\right)}$. The symbol E represents the expected value. The output range of the discriminator is designed to be in the range 0~1. According to the loss function, the parameters of the discriminator are modified to distinguish real data *x* and synthetic data G(z), and, to deceive the discriminator, the parameters of the generator are modified to forge data G(z) that appear real.

### 2.6. Pix2pix

Pix2pix is one of the most powerful deep-learning models on image translation for two different styles of images. It is an extended topology of GANs but has a more complex structure. A pix2pix contains a PatchGAN classifier and u-net. The functions of the PatchGAN classifier and u-net are similar to the discriminator and generator, respectively. A normal (or traditional) classifier maps an image onto a single number. In contrast to the normal classifier, the PatchGAN classifier maps an input image onto a M×M patch, and every element in this patch has its own receptive field from the input image. To do so, the weight number of the classifier can be reduced. It is not necessary to consider the whole input image at a time. The topology of u-net is similar to an autoencoder. The major difference in topology is that u-net has a shortcut between the encoder and decoder but an autoencoder does not. The feature map of the encoder is passed through the shortcut and then concatenated to the feature map of the decoder. Both of them have a bottleneck in the middle of the structure. From the category aspect, pix2pix belongs to a kind of conditional GAN (cGAN) [21] that needs to be provided an extra condition. According to the original paper, the condition can be any type of data, such as a label of class or image data of a certain modality. The training process restricts the output distribution of the u-net and PatchGAN classifiers. Formula (5) is the loss function of cGAN:

(5) $${ℒ}_{cGAN}\left(G,D\right)=E_{c,x}\left[\log D\left(c,x\right)\right]+E_{c,z}\left[\log\left(1-D\left(c,G\left(c,z\right)\right)\right)\right]$$

where we set the MRI as c and CT as x. An additional loss function (6) is applied to the algorithm to enhance sharpness:

(6) $${ℒ}_{L1}\left(G\right)=E_{c,x,z}\left[∥x-G{(c,z)∥}_{1}\right]$$

Therefore, the goal of the algorithm is shown in (7):

(7) $$G^{*}=arg\;\underset{G}{\min}\;\underset{D}{\max}\;{ℒ}_{cGAN}\left(G,D\right)+\lambda {ℒ}_{L1}\left(G\right)$$

where we set the value of λ to 100. We modified the framework from 2D to 3D to prevent slices from forming discontinuities on pseudoimages. In addition, we also modified hyperparameters for the PatchGAN classifier and u-net, such as the learning rate, to match our dataset.

### 2.7. Implementation

**Figure 3:**
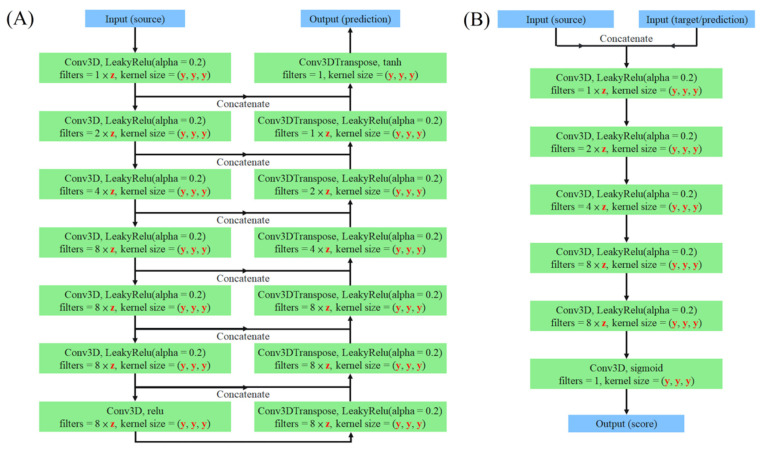
The structure of 3D pix2pix model. A single pix2pix model contains the (**A**) u-net and a (**B**) PatchGAN classifier. The task of u-net is to learn the relationship between “source” and “target” and make the distribution of “prediction” approach the distribution of “target” as similarly as possible. In contrast to u-net, the PatchGAN classifier needs to learn how to cause a high score for the real pair of “target” and “source” rather than the pseudopair of “target” and “prediction”.

We use an open source API, TensorFlow 2.4.1, for deep-learning model-building training and testing. The algorithm is deployed on and executed by the server ESC8000 G4 with a GeForce 1080 Ti (Nvidia, Santa Clara, CA, USA). Figure 3 shows the main architecture of a single model used for every patched dataset. The kernel size is denoted as ky, which means that the kernel size is y × y × y. The base number of filters (or kernel) is denoted as fz, which means that the base number of filters is z. We have three combinations of the number of filters and kernel size, which are denoted as k4_f80, k6_f60, and k8_f30. Consequently, we have 15 (5 patches × 3 filter numbers and kernel size) specific models. For example, the model that has an 80 base filter number and 4 × 4 × 4 kernel size, being trained by the p1 dataset, is denoted as the p1_k4_f80 model. In the algorithm, the stride of 3D convolution and transpose convolution is (2, 2, 2), and the batch size is 1.

### 2.8. Merge Prediction from the k4_f80/k6_f60/k8_f80 Model

**Figure 4:**
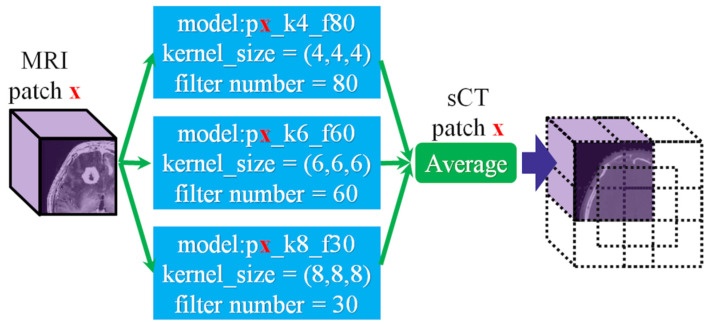
Merging prediction from the k4_f80, k6_f60, and k8_f30 models. A patch of synthetic CT is composed of output from the k4_f80, k6_f60, and k8_f30 models.

The kernel size is an important factor in convolutional neural networks [22,23,24]. In general, a model with a small kernel is more efficient, and a large kernel is more accurate. The element in the feature map has its own receptive field, which is related to kernel size. That is, if we choose different kernel sizes, it results in obtaining different feature maps. Although a larger kernel considers more information from the former layer to produce feature maps at a time, a larger kernel does not present better performance as long as the kernel size is over a certain number. As shown in Figure 4, we mixed 3 outputs of well-trained models for a single patched dataset. The base kernel sizes of these 3 well-trained models are 4 × 4 × 4, 6 × 6 × 6, and 8 × 8 × 8. The proportion of ingredients of the mixed image is 1:1:1.

### 2.9. Merge Prediction from the p1~p5 Model

To eliminate discontinuous boundaries that appear at the merged prediction, the center locations of each patch in the re-FOV image are designed such that there is overlap among these patches. We build and train 5 specific models, and each model is specialized for the corresponding patched dataset. After the training process, we create an all-zero image, called the base map, with a size of 200 × 200 × 128. The output value of all patched models is linearly converted to the same output range as the p3 model. Then, based on the location of each specific patch at the re-FOV image, the prediction of each specific model is added back to the base map. Every voxel in the base map is divided by the number of overlapping times. The numbered marks of the re-FOV image in Figure 2 present the amount of overlap in each space.

### 2.10. Image Similarity Evaluation

The two images *A* and *B* are given and shown in (8):

(8) $$A=\left[\begin{array}{c} a_{1}\\ a_{2}\\ \vdots \\ a_{N} \end{array}\right],\;B=\left[\begin{array}{c} b_{1}\\ b_{2}\\ \vdots \\ b_{N} \end{array}\right]$$

where $a_{1}$, $a_{2}$, …… and $a_{N}$ are the voxels in image *A*, and $b_{1}$, $b_{2}$, …… and $b_{N}$ are the voxels in image *B*. To objectively determine the difference between images *A* and *B*, we adopt the quantized index for evaluation. Formulas (9)–(11) are used to calculate the cosine angle distance (CAD) [25,26], L2 norm (also known as Euclidean distance) [25,27], and mean structural similarity index (MSSIM) [28], respectively.

CAD:

(9) $$CAD\left(A,\;B\right)=\cos\left(\theta \right)=\frac{A\cdot B}{∥A∥\times ∥B∥}$$

L2 norm:

(10) $$L2\left(A,\;B\right)=\sqrt{\sum_{i=1}^{N}{\left(a_{i}-b_{i}\right)}^{2}}$$

MSSIM:

(11) $$\left\{\begin{array}{c} SSIM\left(A_{i}^{\prime },B_{i}^{\prime }\right)={\left[l\left(A_{i}^{\prime },B_{i}^{\prime }\right)\right]}^{\alpha }{\left[c\left(A_{i}^{\prime },B_{i}^{\prime }\right)\right]}^{\beta }{\left[s\left(A_{i}^{\prime },B_{i}^{\prime }\right)\right]}^{\gamma }\\ l\left(A_{i}^{\prime },B_{i}^{\prime }\right)=\frac{2\mu_{A_{i}^{\prime }}\mu_{B_{i}^{\prime }}+C_{1}}{{\left(\mu_{A_{i}^{\prime }}\right)}^{2}+{\left(\mu_{B_{i}^{\prime }}\right)}^{2}+C_{1}}\\ c\left(A_{i}^{\prime },B_{i}^{\prime }\right)=\frac{2\sigma_{A_{i}^{\prime }}\sigma_{B_{i}^{\prime }}+C_{2}}{{\left(\sigma_{A_{i}^{\prime }}\right)}^{2}+{\left(\sigma_{B_{i}^{\prime }}\right)}^{2}+C_{2}}\\ s\left(A_{i}^{\prime },B_{i}^{\prime }\right)=\frac{\sigma_{A_{i}^{\prime }B_{i}^{\prime }}+C_{3}}{\sigma_{A_{i}^{\prime }\sigma_{B_{i}^{\prime }}+C_{3}}}\\ w=\left\{w_{i}|i=1,2,\ldots \ldots ,N\right\}\\ \mu_{A_{i}^{\prime }}=\overset{N}{\underset{j=1}{\sum }}w_{j}a_{i,j}^{\prime },\mu_{B_{i}^{\prime }}=\overset{N}{\underset{j=1}{\sum }}w_{j}b_{i,j}^{\prime }\\ \sigma_{A_{i}^{\prime }}=\sqrt{\sum_{j=1}^{N}w_{j}}{\left(a_{i,j}^{\prime }-\mu_{A_{i}^{\prime }}\right)}^{2},\;\sigma_{B_{i}^{\prime }}=\sqrt{\sum_{j=1}^{N}w_{j}{\left(b_{i,j}^{\prime }-\mu_{B_{i}^{\prime }}\right)}^{2}}\\ \sigma_{A_{i}^{\prime }B_{i}^{\prime }}=\overset{N}{\underset{j=1}{\sum }}w_{j}\left(a_{i,j}^{\prime }-\mu_{A_{i}^{\prime }}\right)\left(b_{i,j}^{\prime }-\mu_{B_{i}^{\prime }}\right)\\ MSSIM\left(A,B\right)=\frac{1}{M}\overset{M}{\underset{i=1}{\sum }}\mathrm{SSIM}\left(A_{i}^{\prime },B_{i}^{\prime }\right) \end{array}\right.$$

When images A and B are the same, the values of the CAD, L2 norm, and MSSIM are 1, 0, and 1, respectively. These similarity indices provide quantized values and different aspects to evaluate two images. Images A and B are treated as two vectors when the value of CAD is calculated. The angle between these two vectors shows whether image A is similar to image B. The value of the L2 norm value represents the accumulation comparison for voxelwise differences of images *A* and B. The MSSIM considers three important factors: luminance, contrast, and structure. In Formula (11), M is the number of local patches in full image A. The i-th patch $A_{i}^{\prime }$ of image A contains voxels $a_{i,1}^{\prime }$, $a_{i,2}^{\prime }$, ……, $a_{i,N}^{\prime }$; w is a circular-symmetric Gaussian weighting function with a standard deviation of 1.5 samples, normalized to the unit sum $\left(\overset{N}{\underset{i=1}{\sum }}w_{i}=1\right)$. We take advantage of scikit-image, which is a collection of algorithms for image processing, to calculate MSSIM. It is the function “skimage.metrics.structural_similarity()” that we use, leaving all the parameters as default.

### 2.11. Clinical Evaluation

Two radiologists independently evaluated the satisfaction score, including spatial, detail, contrast, noise, and artifacts, and categorized them into excellent, good, fair, or bad for each imaging attribute. Reader agreement regarding invasion depth was analyzed using weighted kappa statistics (0.00 ≤ k < 0.40 indicated poor agreement; 0.40 ≤ k ≤ 0.70 indicated fair agreement; k > 0.70 indicated excellent agreement). The Mann–Whitney U test was used to compare the clinical satisfaction scores between the bone and soft tissue window images from the synthetic CT.

## 3. Result

**Figure 5:**
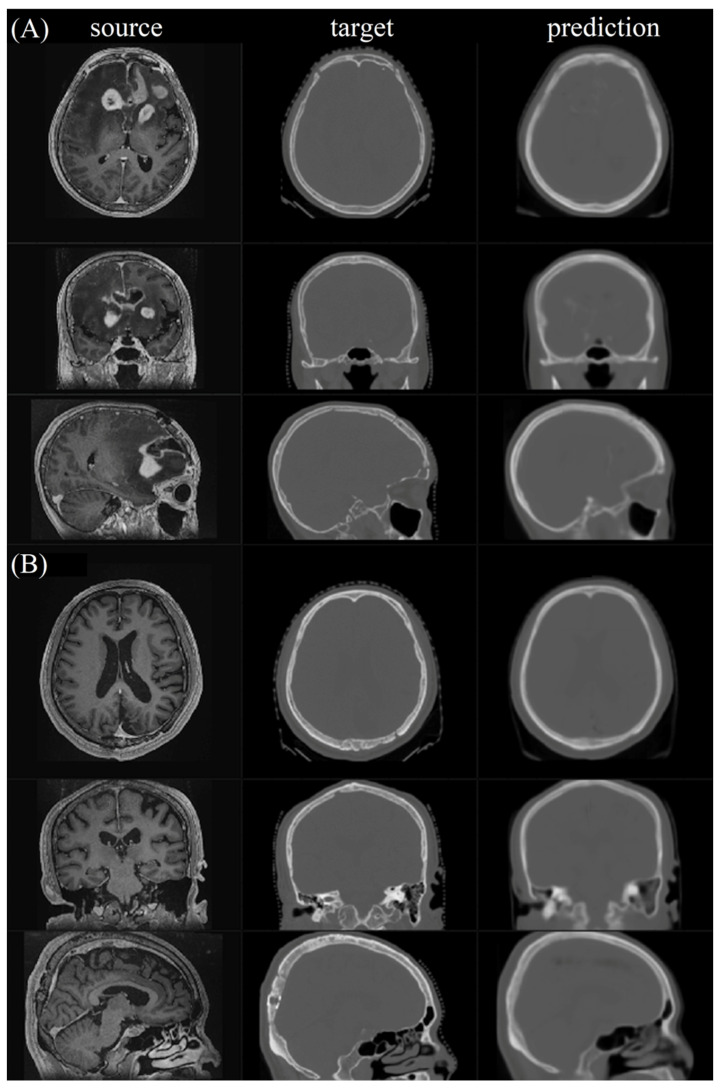
The final merged prediction. (**A**,**B**) show the two testing pairs, which are No. 001 and No. 003, and their corresponding merged predictions, respectively.

**Figure 6:**
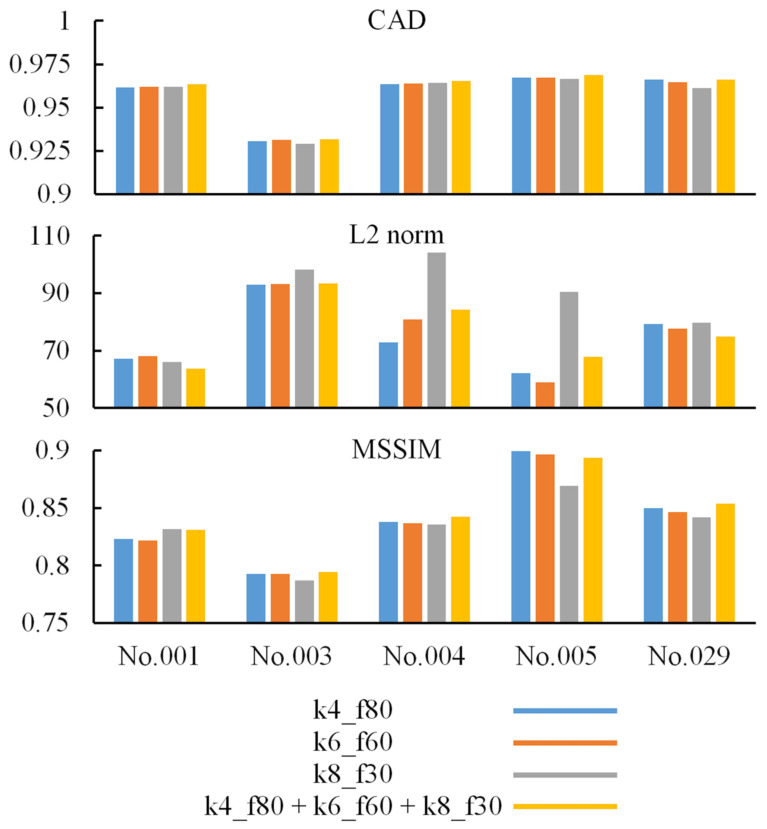
The individual similarity indices before and after merging. To compare the contributions of different kernel sizes, the calculations of similarity indices are performed according to kernel size. The entire synthetic CT evaluated by the indices is made of outputs of p1~p5 models.

Figure 5 shows the first two testing pairs and their synthetic CT. Each merged prediction is composed of outputs from 15 models. The models of participation are p1_k4_f80, p2_k4_f80, p3_k4_f80, p4_k4_f80, p5_k4_f80, p1_k6_f50, p2_k6_f50, p3_k6_f50, p4_k6_f50, p5_k6_f50, p1_k8_f30, p2_k8_f30, p3_k8_f30, p4_k8_f30, and p5_k8_f30. Because the algorithm is based on a 3D framework, we plot axial, coronal, and sagittal views for each sample. The similarity indices for all tested CT scans and their corresponding synthetic CT scans are shown in Figure 6. The additional two similarity indices, MSE and PSNR, of CT vs. synthetic CT before and after merging were calculated to evaluate the model performance (Supplementary Materials Table S3).

**Figure 7:**
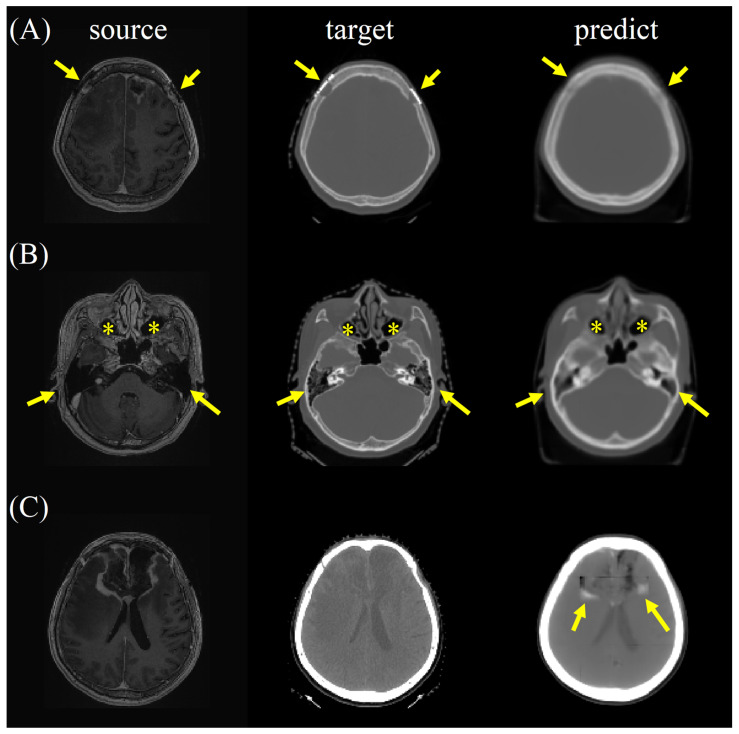
(**A**) The metallic artifact was reduced (arrows), and (**B**) the air density of the paranasal sinuses (asterisks) and mastoid air cells (arrows) were well preserved on the bone window images of the synthetic CT. (**C**) Perifocal hyperdensities (arrows) on the soft tissue window images of the synthetic CT might lead to a false impression of intracranial hemorrhage, which should have been postoperative encephalomalacia and white matter edema.

For synthetic CT, radiologists valued excellent satisfaction in spatial geometry and noise level, good satisfaction in contrast and artifacts, and fair imaging details for the bone and soft tissue window images (Supplementary Materials Tables S4 and S5). Higher satisfaction scores were observed on the bone window images than on the soft tissue window images for evaluation of the details, contrast, and artifact (Table 1). There was no statistically significant difference in synthetic CT on axial, coronal, or sagittal planes. The reader agreement rate was excellent in terms of spatial, detail, contrast, noise, and artifact in axial, coronal, and sagittal planes for both the bone and soft tissue window images from the synthetic CT. Interestingly, the metallic artifact was reduced, and the air density of the paranasal sinuses and mastoid air cells were well preserved on the bone window images of the synthetic CT (Figure 7a,b). Of note, perifocal hyperdensities on the soft tissue window images of the synthetic CT might lead to a false impression of intracranial hemorrhage, which should have been postoperative encephalomalacia and white matter edema (Figure 7c).

## 4. Discussion

### 4.1. Acquiring Paired Data

Paired data are essential for training models in 3D pix2pix, and reports have shown the feasibility of 3D pix2pix in synthetic images from multiparametric MRI. One novelty of this study is synthetic CT from contrast-enhanced MRI is important for radiotherapy treatment planning. However, the amount of treatment planning data cannot be comparable to diagnostic CT or MRI. Therefore, we developed a preprocessing pipeline to overcome different imaging modalities with different voxel sizes (resolutions), FOVs, window widths, and levels of intensity. Furthermore, data augmentation was established to expand the utility of sparse clinical imaging data. Our process involved little human involvement and is believed to be scalable to a larger dataset.

### 4.2. Effect of Data Augmentation

**Figure 8:**
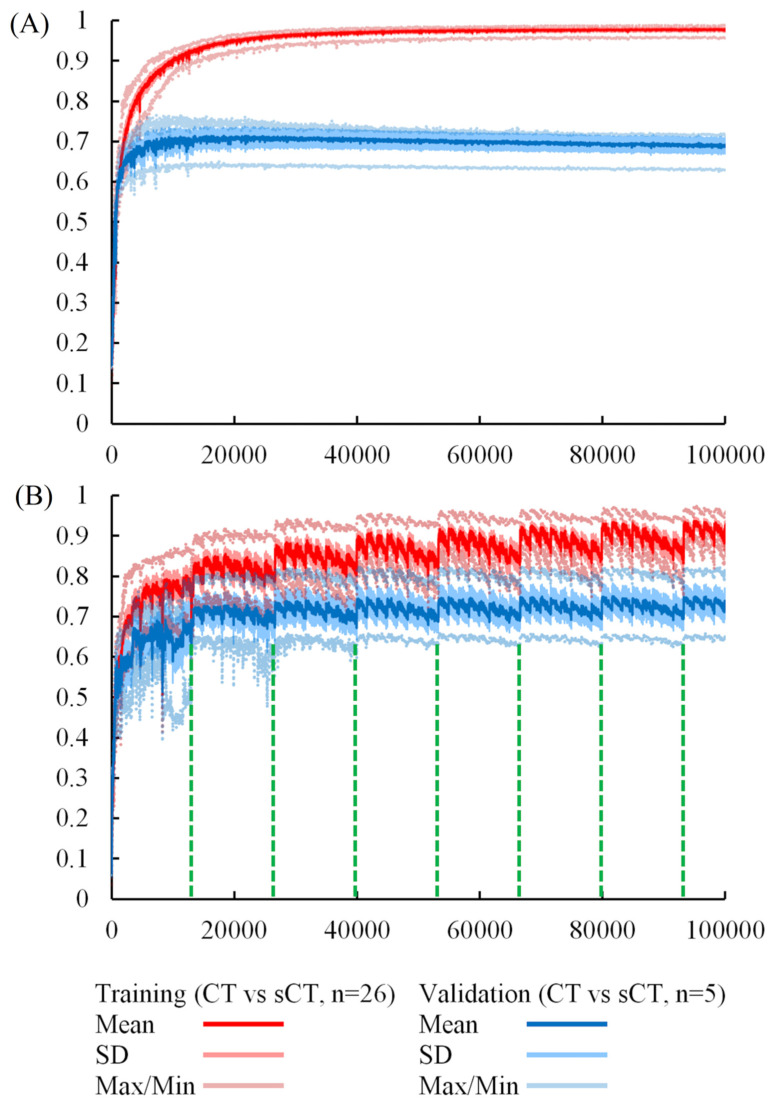
The MSSIM of training process. (**A**,**B**) show the results of Experiment 1 and Experiment 2, respectively. The performance of the p3_k4_f80 model is validated with original training data (*n* = 26) and testing data (*n* = 5) after 26 iterations. One epoch equivalent to 26 iterations and the period of data augmentation is 13,312 iterations (26 × 512). The data augmentation cycles end at iterations 13,312; 26,624; 39,936; 53,248; 66,560; 79,872; and 93,184. “SD” is the abbreviation for “standard deviation”.

Although several studies [18,19,20] have indicated that data augmentation is a useful solution for limited data, none of them focused on the 3D image translation field. To ensure the effect of data augmentation for our models, we performed two simple experiments. A model was trained by a dataset without augmentation in Experiment 1. In Experiment 2, a model was trained by an augmented dataset wherein the voxel coordinates of all images were rotated by a different angle once at the beginning of each epoch. All the other conditions are the same for Experiments 1 and 2. Figure 8 shows the MSSIM trends of the two experiments.

The trend of Figure 8 implies that data augmentation indeed prevents a model from overfitting. It is obvious that the model overfits seriously at the first beginning in Experiment 1, and the growth of the mean MSSIM score of the testing data stops at approximately 0.71. Moreover, we inspected every synthetic CT from Experiment 1, and it is obvious that the image was blurry and unclear. On the other hand, the overfitting phenomena of the other experiments improved quite well. The best mean MSSIM scores are breakthroughs over 0.75. Furthermore, according to the slope of the curve, the index is still growing after 100,000 iterations. Therefore, the data augmentation technique we proposed does increase feature variety, which is helpful for model generalization.

### 4.3. The Approach Is Extendable for Higher-Resolution Datasets

In this paper, we obtain five patched datasets from the original dataset wherein each image has a very high number of voxels. It is impossible to create a model to manage our original dataset directly, and the number of trainable parameters is too numerous to be affordable for hardware. Compared to the original dataset, the benefit of a patched dataset is that it has fewer features and fewer voxels. Even if we take advantage of patched datasets to shrink the model, the number of trainable parameters of the model is approximately 300~400 million in this work for a single model. We think MR of the same body part among different patients has similar features, as does CT. Therefore, it is rational to build and train a specific model for a specific patched dataset that contains the MRI and CT of the same body part from all subjects. The solution can be adopted on any other paired dataset that has higher resolutions as long as the dataset is patched into smaller parts.

### 4.4. Demand for Pair Data

The 3D pix2pix model can learn the existing relationship between two imaging modalities from a training dataset as long as the relationship is certain. However, there are several uncertain relationships within our paired dataset. The material itself is the main cause of uncertain relationships. Some of the subjects had metal dentures, but some did not. Both metal dentures and normal teeth form a bright area on CT images, whereas they can hardly be seen on MR images. The shape of soft tissue and posture of a patient are also common causes of uncertain relationships. A patient might face a different angle when taking a brain MRI and CT exam or have a different bladder shape when taking a pelvis MRI and CT exam. The resolution of the imaging modality itself is another cause of uncertainty, and a medical image might be affected by the partial volume effect. In addition, MR and CT imaging have different resolutions in our original dataset, and the information amounts (voxel numbers) of the two modalities in space are not equal. Even if the dataset is resampled, some information might be lost during the process or cannot reflect reality. Some of the other uncertain relationships are caused by phantoms, noise, distortion, and inaccurate image registration.

### 4.5. Effect of Multiple Kernel Sizes

In Figure 6, most indices based on models with multiple kernel sizes are higher than the indices based on models with a single kernel size. We infer that it is helpful to improve prediction by merging output from different models with multiple kernel sizes. The reason why some index values are not the highest on the comparison is affected by the uncertain relationship of testing paired data. Because of the uncertainty mentioned above, some synthetic CT scans are more correct than the corresponding real CT scans (i.e., target or ground truth) on the shape or the position. Therefore, the value of similarity indices not only implies the correctness of prediction but also implicitly presents uncertainty in paired data.

### 4.6. Performance Variations between Kernels

The manner of data preprocessing affects the performance variances most deeply. For our early stage attempt, the composite image possesses mosaic style. Some predicted areas are bright and others are dark. The boundary between different predicted patch areas is quite sharp. The reason for performance variation is that we split the dataset into five patched datasets before performing normalization and standardization. The normalization and standardization are based on every patched dataset itself. Therefore, the five patched datasets have no common baseline. We made several tries in order to eliminate the performance variations, such as controlling the input training data originating from the same people for the five patched models that have the same iterations. Compared to the previous version of the process, the final version improves the issue considerably. The performance variations of the proposed version are tiny. The other important factor is the native dataset itself. We found that the p3 model is the most difficult one to train and its performance is slightly poorer than all the other models. The rational explanation is that the image FOV in patched dataset 3 is allocated at the center of the brain. Thus, the patched dataset 3 contains a larger number of complicated features.

### 4.7. Limitation

The algorithm belongs to supervised learning, and a paired dataset is needed. The p1~p5 models are trained independently such that the phenomenon of grid-like boundary or nonuniform luminance appears in the final compound image. The drawback of expandable methodology is that cost of storage capacity and the quantities of calculation for training and prediction are proportional to the number of patches. The data form in the dataset makes the size of the dataset is difficult to enlarge. In the future, we hope the CT datasets generated from diagnostic MRIs can be used directly for radiation planning. The electron densities are the basis of the work of the irradiation planning programs. Therefore, the comparison of calculated synthetic electron densities and electron densities of the real CT scans will be addressed in the future.

## 5. Conclusions

In this paper, we propose an extensible solution for 3D image translation with a high-resolution dataset and demonstrate the effectiveness of data augmentation under the circumstance of insufficient training data. With the 3D image patching technique, the model size is no longer a major obstacle for the hardware. A set of well-trained models are capable of converting MRI to CT, which provides helpful information for clinical examination and reduces the radiation damage of CT imaging. For synthetic CT, radiologists reported excellent satisfaction in spatial geometry and noise level, good satisfaction in contrast and artifacts, and fair imaging details. There was no statistically significant difference in synthetic CT on the axial, coronal, or sagittal planes. The present project presents a pivotal role in connecting the MRI and CT datasets, and the application could be expandable from the cranium to other body parts. We will focus on eliminating the clinical testing difference between synthetic CT and real CT in the future.

## Figures and Tables

**Table 1:** Clinical satisfaction score based on the bone and soft tissue window synthetic CT.

	Case	Bone		Soft Tissue		*p*
		Median	Range	Median	Range	
AXL	spatial	4	3–4	4	3–4	0.71
	detail	2	2–3	1	1–2	<0.001
	contrast	4	2–4	3	3–4	<0.001
	noise	4	3–4	4	3–4	0.88
	artifact	3	1–4	3	2–4	<0.001
COR	spatial	4	3–4	4	3–4	1.00
	detail	2	2–3	2	1–2	<0.001
	contrast	4	2–4	3	3–4	<0.001
	noise	4	4–4	4	3–4	0.32
	artifact	4	3–4	4	2–4	<0.001
SAG	spatial	4	3–4	4	3–4	1.00
	detail	2	2–3	2	1–2	<0.001
	contrast	4	3–4	3	3–4	<0.001
	noise	4	3–4	4	3–4	1.00
	artifact	4	3–4	3	2–4	<0.001

## Data Availability

Due to the ethical approval and requirements of the data protection legislation, the data set will only be made available on a restricted basis according to the data sharing policies at the Chang Gung Memorial Hospital at Linkou, Taoyuan, Taiwan. Applications for access to anonymized data can be obtained by sending an e-mail to jcweng@mail.cgu.edu.tw.

## Supplementary Materials

The following supporting information can be downloaded at: https://www.mdpi.com/article/10.3390/jpm12030361/s1, Table S1: The detail of original dataset. The table lists the voxel numbers and voxel size for each pairs in the original dataset; Table S2: The modified image parameters after reFOV and resampling for few more subjects from training and test data; Table S3: The additional similarity indices for CT vs synthetic CT before and after merge. The other two similarity indices, MSE and PSNR, are calculated for evaluation of model performance; Table S4: Clinical satisfaction score based on the bone window synthetic CT.
