# Supplementary material for: Magnetic Resonance-Based Synthetic Computed Tomography Using Generative Adversarial Networks for Intracranial Tumor Radiotherapy Treatment Planning
Source: J Pers Med. 2022 Feb 26;12(3):361. doi: 10.3390/jpm12030361 (PMC8955512; doi:10.3390/jpm12030361)
Supplement: Supplementary file 1 [file jpm-12-00361-s001.zip › jpm-1584745-supplementary (1).pdf]

Supporting Materials Table S1. The detail of original dataset. The table lists the voxel numbers and voxel size for each pairs in the original dataset.

type	filename	Voxel number	W, H, D (mm)	Voxel size (mm)
Training	CT_002	512 × 512 × 361	500, 500, 451.25	0.98 × 0.98 × 1.25
	MR_002	512 × 512 × 150	256, 256, 150	0.5 × 0.5 × 1
	CT_006	512 × 512 × 361	500, 500, 451.25	0.98 × 0.98 × 1.25
	MR_006	512 × 512 × 158	256, 256, 158	0.5 × 0.5 × 1
	CT_007	512 × 512 × 361	500, 500, 451.25	0.98 × 0.98 × 1.25
	MR_007	512 × 512 × 172	220, 220, 172	0.43 × 0.43 × 1
	CT_008	512 × 512 × 361	500, 500, 451.25	0.98 × 0.98 × 1.25
	MR_008	512 × 512 × 166	256, 256, 166	0.5 × 0.5 × 1
	CT_009	512 × 512 × 361	500, 500, 451.25	0.98 × 0.98 × 1.25
	MR_009	512 × 512 × 190	256, 256, 190	0.5 × 0.5 × 1
	CT_010	512 × 512 × 361	500, 500, 451.25	0.98 × 0.98 × 1.25
	MR_010	512 × 512 × 180	256, 256, 180	0.5 × 0.5 × 1
	CT_011	512 × 512 × 361	500, 500, 451.25	0.98 × 0.98 × 1.25
	MR_011	512 × 512 × 136	220, 220, 136	0.43 × 0.43 × 1
	CT_012	512 × 512 × 361	500, 500, 451.25	0.98 × 0.98 × 1.25
	MR_012	512 × 512 × 160	256, 256, 160	0.5 × 0.5 × 1
	CT_013	512 × 512 × 361	500, 500, 451.25	0.98 × 0.98 × 1.25
	MR_013	512 × 512 × 174	256, 256, 174	0.5 × 0.5 × 1
	CT_014	512 × 512 × 361	500, 500, 451.25	0.98 × 0.98 × 1.25

	MR_014	512 × 512 × 162	256, 256, 162	0.5 × 0.5 × 1
	CT_015	512 × 512 × 361	500, 500, 451.25	0.98 × 0.98 × 1.25
	MR_015	512 × 512 × 162	256, 256, 162	0.5 × 0.5 × 1
	CT_016	512 × 512 × 361	500, 500, 451.25	0.98 × 0.98 × 1.25
	MR_016	512 × 512 × 180	256, 256, 180	0.5 × 0.5 × 1
	CT_017	512 × 512 × 361	500, 500, 451.25	0.98 × 0.98 × 1.25
	MR_017	512 × 512 × 150	200, 200, 150	0.39 × 0.39 × 1
	CT_018	512 × 512 × 361	500,500,451.25	0.98 × 0.98 × 1.25
	MR_018	512 × 512 × 174	256, 256, 174	0.5 × 0.5 × 1
	CT_019	512 × 512 × 361	500,500,451.25	0.98 × 0.98 × 1.25
	MR_019	512 × 512 × 168	210, 210, 168	0.41 × 0.41 × 1
	CT_020	512 × 512 × 361	500,500,451.25	0.98 × 0.98 × 1.25
	MR_020	512 × 512 × 162	256, 256, 162	0.5 × 0.5 × 1
	CT_021	512 × 512 × 361	500,500,451.25	0.98 × 0.98 × 1.25
	MR_021	512 × 512 × 172	256, 256, 172	0.5 × 0.5 × 1
	CT_022	512 × 512 × 361	500,500,451.25	0.98 × 0.98 × 1.25
	MR_022	512 × 512 × 174	220, 220, 174	0.43 × 0.43 × 1
	CT_023	512 × 512 × 361	500,500,451.25	0.98 × 0.98 × 1.25
	MR_023	512 × 512 × 142	256, 256, 142	0.5 × 0.5 × 1
	CT_024	512 × 512 × 361	500, 500, 451.25	0.98 × 0.98 × 1.25
	MR_024	512 × 512 × 160	256, 256, 160	0.5 × 0.5 × 1
	CT_025	512 × 512 × 361	500, 500,	0.98 × 0.98 ×

			451.25	1.25
	MR_025	512 × 512 × 182	256, 256, 182	0.5 × 0.5 × 1
	CT_026	512 × 512 × 361	500, 500, 451.25	0.98 × 0.98 × 1.25
	MR_026	512 × 512 × 146	256, 256, 146	0.5 × 0.5 × 1
	CT_027	512 × 512 × 361	500, 500, 451.25	0.98 × 0.98 × 1.25
	MR_027	512 × 512 × 164	256, 256, 164	0.5 × 0.5 × 1
	CT_028	512 × 512 × 361	500, 500, 451.25	0.98 × 0.98 × 1.25
	MR_028	512 × 512 × 172	210, 210, 172	0.41 × 0.41 × 1
	CT_030	512 × 512 × 361	500, 500, 451.25	0.98 × 0.98 × 1.25
	MR_030	512 × 512 × 166	256, 256, 166	0.5 × 0.5 × 1
	CT_031	512 × 512 × 361	500, 500, 451.25	0.98 × 0.98 × 1.25
	MR_031	512 × 512 × 176	256, 256, 176	0.5 × 0.5 × 1
Testing	CT_001	512 × 512 × 361	500, 500, 451.25	0.98 × 0.98 × 1.25
	MR_001	512 × 512 × 150	256, 256, 150	0.5 × 0.5 × 1
	CT_003	512 × 512 × 361	500, 500, 451.25	0.98 × 0.98 × 1.25
	MR_003	512 × 512 × 160	256, 256, 160	0.5 × 0.5 × 1
	CT_004	512 × 512 × 361	500, 500, 451.25	0.98 × 0.98 × 1.25
	MR_004	512 × 512 × 158	256, 256, 158	0.5 × 0.5 × 1
	CT_005	512 × 512 × 361	500, 500, 451.25	0.98 × 0.98 × 1.25
	MR_005	512 × 512 × 162	200, 200, 162	0.39 × 0.39 × 1
	CT_029	512 × 512 × 361	500, 500, 451.25	0.98 × 0.98 × 1.25
	MR_029	512 × 512 × 168	256, 256, 168	0.5 × 0.5 × 1

Supporting Materials Table S2. The additional similarity indices for CT vs synthetic CT before and after merge. The other two similarity indices, MSE and PSNR, are calculated for evaluation of model performance.

Model	No.	CAD	L2 norm	MSE	PSNR	MSSIM
k4_f80	001	0.96163	67.17678	0.00088	30.54832	0.82331
	003	0.93081	92.96263	0.00169	27.72653	0.79258
	004	0.96347	72.93508	0.00104	29.83397	0.83787
	005	0.96717	62.23005	0.00076	31.21270	0.89937
	029	0.96633	79.33760	0.00123	29.10312	0.84987
k6_f60	001	0.96212	68.14185	0.00091	30.42442	0.82177
	003	0.93133	93.13833	0.00169	27.71013	0.79287
	004	0.96404	80.76704	0.00127	28.94802	0.83683
	005	0.96713	59.00892	0.00068	31.67435	0.89678
	029	0.96465	77.60409	0.00118	29.29501	0.84654
k8_f30	001	0.96201	66.09462	0.00085	30.68938	0.83142
	003	0.92913	98.22321	0.00188	27.24842	0.78697
	004	0.96431	104.02715	0.00211	26.74977	0.83547
	005	0.96654	90.45831	0.00160	27.96373	0.86928
	029	0.96119	79.66863	0.00124	29.06695	0.84208
k4_f80 + k6_f60 + k8_f30	001	0.96368	63.77412	0.00079	30.99981	0.83082
	003	0.93187	93.30764	0.00170	27.69436	0.79472
	004	0.96533	84.33236	0.00139	28.57281	0.84223
	005	0.96871	67.89179	0.00090	30.45636	0.89400
	029	0.96623	74.86371	0.00109	29.60727	0.85412

Supporting Materials Table S3. Clinical satisfaction score based on the bone window synthetic CT

	case	I	II	III	IV	V
	spatial	4	4	3.992	4	4
	detail	2	2	2	2	2
	contrast	3.697	3.992	3.984	4	3.484
	noise	4	4	3.938	4	4
AXL	artifact	3.533	3.625	3.438	3.143	2.961
	spatial	4	4	4	4	4
	detail	2	2	2	2	2
	contrast	3.827	3.763	3.844	3.908	3.578
	noise	4	4	4	4	4
COR	artifact	3.846	4	4	3.693	3.565
	spatial	4	4	4	4	4
	detail	2	2	2	2	2
	contrast	3.719	3.966	4	4	3.812
	noise	4	4	4	4	4
SAG	artifact	3.933	3.818	3.986	3.566	3.292

Supporting Materials Table S4. Clinical satisfaction score based on the softy tissue window synthetic CT

	case	I	II	III	IV	V
	spatial	4	4	4	4	4
	detail	1.333	1.602	1.512	1.270	1.234
	contrast	3	3	3	3	3
	noise	4	4	3.944	4	4
AXL	artifact	2.925	3.156	3.119	3.317	2.961
	spatial	4	4	4	4	4
	detail	1.713	1.651	1.346	1.255	1.882
	contrast	3	3	3	3	3
	noise	4	4	4	4	4
COR	artifact	3.449	3.846	3.270	3.693	3.255
	spatial	4	4	4	4	4
	detail	1.652	1.709	1.350	1.154	1.758
	contrast	3	3	3	3	3
	noise	4	4	4	4	4
SAG	artifact	3.385	3.216	3.615	3.531	3.240
